# Treatment of Bleeding Episodes With Efanesoctocog Alfa in Previously Treated Patients With Severe Hemophilia A in the Phase 3 XTEND‐1 Study

**DOI:** 10.1002/ajh.27603

**Published:** 2025-02-10

**Authors:** Angela C. Weyand, Sandrine Meunier, Nobuaki Suzuki, Linda Bystrická, Graham Neill, Lydia Abad‐Franch, Annemieke Willemze, Alberto Tosetto

**Affiliations:** ^1^ Division of Hematology/Oncology, Department of Pediatrics University of Michigan Ann Arbor Michigan USA; ^2^ Hospices Civils de Lyon Groupement Hospitalier Universitaire Est, Unité Hémostase Clinique Bron France; ^3^ Department of Transfusion Medicine Nagoya University Hospital Aichi Japan; ^4^ Sobi Basel Switzerland; ^5^ Sanofi Reading UK; ^6^ Sanofi Amsterdam Netherlands; ^7^ Hemophilia and Thrombosis Center Hematology Department, San Bortolo Hospital Vicenza Italy

## Abstract

Despite therapeutic advances, people with hemophilia experience bleeds. These may be life‐threatening, result in permanent joint damage, chronic pain, difficulties with mobility/daily activities, and impact quality of life. In the XTEND‐1 study (NCT04161495), once‐weekly efanesoctocog alfa (50 IU/kg) prophylaxis provided highly effective bleed prevention and high‐sustained factor levels for most of the week and was well‐tolerated. We report post hoc analysis of bleeding episodes and their treatment in previously treated patients (≥ 12 years old). Participants received 50 IU/kg efanesoctocog alfa either as once‐weekly prophylaxis (Arm A) or on‐demand followed by once‐weekly prophylaxis (Arm B) in XTEND‐1. Endpoints included treatment of bleeding episodes and response to treatment. During XTEND‐1, 422 bleeding episodes were reported among 159 participants; 362 were treated. Most treated bleeding episodes (74%; *n* = 268) occurred during the Arm B on‐demand period, of which 197 (74%) were spontaneous. Seventy‐five participants had no bleeding episodes in Arm A; all in Arm B had ≥ 1 bleeding episode while on‐demand. Most participants (*n* = 107, 81%) had zero treated spontaneous bleeding episodes and rates of treated bleeding episodes in Arm A (prophylaxis) were low (median [interquartile range] overall ABR: 0.00 [0.00–1.04]). A single injection was sufficient to resolve 97% (350/362) of treated bleeding episodes, no bleeding episodes required > 3 injections, and responses to 95% of evaluable injections were rated excellent/good. Median total dose was 50.9 IU/kg per bleeding episode. Results of this analysis further demonstrated that once‐weekly efanesoctocog alfa provides highly effective bleed protection and treatment of bleeding episodes in participants with severe hemophilia A.

**Trial Registration:** NCT04161495

## Graphical abstract

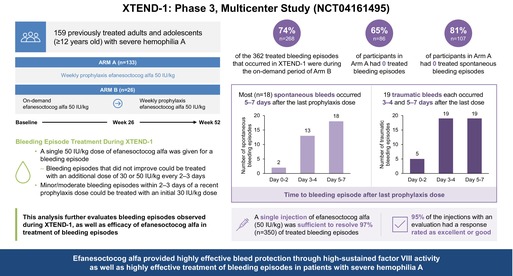

## 1 Introduction

People with severe hemophilia commonly experience bleeding episodes in the joints, muscles, and internal organs, which range in severity and may be life‐threatening; up to 80% of bleeding episodes occur in the joints [1]. Bleeding, particularly intracranial and gastrointestinal, is still a leading cause of death in people with hemophilia [1, 2, 3]. Recurrent and suboptimally controlled joint bleeds can lead to poor long‐term outcomes including permanent joint damage [1, 4, 5, 6]. The long‐term impact of poor bleed control also includes substantial detrimental effects on patient quality of life, including difficulties with mobility, daily activities, and an association with increased pain and discomfort [7]. Patients with long‐term sequelae also report higher anxiety and depression compared to those without [7].

Prophylactic treatment initiation early in life is recommended to prevent musculoskeletal complications from recurrent joint and muscle bleeds [1]. While prophylaxis with factor VIII (FVIII) replacement products significantly decreases the risk of bleeding episodes and improves clinical outcomes, existing standard half‐life (SHL) and extended half‐life (EHL) FVIII products are associated with significant treatment burdens related to the frequent intravenous administration (several times a week or every other day) required to maintain FVIII levels > 1 IU/dL [1, 8, 9, 10]. Furthermore, even if factor trough levels of 1–3 IU/dL are achieved with existing FVIII products, this may still be suboptimal for total prevention of bleeds [1, 10]; factor levels of up to 35% may be required for near‐zero joint bleed rates [11, 12, 13]. While the standard of care for all patients with severe hemophilia A is FVIII replacement therapy with clotting factor concentrates, or other hemostatic products, the ability to increase protection from bleeds by achieving higher sustained levels of FVIII activity remains a critical need for patients that is recognized by the World Federation of Hemophilia [1, 9, 14]. Accordingly, there has been an unmet need for FVIII products that can provide high‐sustained factor levels while reducing the treatment burden and improving overall quality of life for patients with hemophilia A.

Efanesoctocog alfa is a first‐in‐class high‐sustained FVIII replacement therapy that decouples recombinant FVIII from endogenous von Willebrand factor (VWF) in circulation, overcoming the VWF‐imposed limits on half‐life [15, 16, 17]. Indeed, efanesoctocog alfa has a 3‐ to 4‐fold longer elimination half‐life than SHL and EHL products [18]. The completed Phase 3 study, XTEND‐1 (NCT04161495), demonstrated that once‐weekly efanesoctocog alfa prophylaxis provided superior bleed prevention compared with prior FVIII prophylaxis in adults and adolescents with severe hemophilia A and was well tolerated [19]. In XTEND‐1, efanesoctocog alfa provided high‐sustained FVIII levels throughout the weekly dosing interval, with mean FVIII levels in the normal to near‐normal range (> 40 IU/dL) for ~4 days post dose, and 15% at Day 7 [19]. Here we present further evaluation of bleeding episodes observed during XTEND‐1, as well as the efficacy of efanesoctocog alfa in the treatment of bleeding episodes during the study.

## 2 Methods

### 2.1 Study Design

XTEND‐1 (NCT04161495) was a Phase 3, open‐label, multicenter study to assess the efficacy and safety of efanesoctocog alfa in previously treated adult and adolescent (≥ 12 years) patients with severe hemophilia A [19]. The study was composed of 2 treatment arms: patients on pre‐study FVIII prophylaxis were enrolled into Arm A where they received 52 weeks of once‐weekly efanesoctocog alfa (50 IU/kg) prophylaxis and patients receiving on‐demand therapy prior to XTEND‐1 were enrolled into Arm B and received 26 weeks of on‐demand efanesoctocog alfa (50 IU/kg), followed by 26 weeks of once‐weekly prophylaxis (50 IU/kg). The study consisted of an up to 8‐week screening period, a 52‐week open‐label treatment period, and a 2–3‐week safety follow‐up period. Most of the participants had completed an observational study prior to enrollment in XTEND‐1 [20]. The study was performed in accordance with the Declaration of Helsinki and all local regulations.

### 2.2 Patient Population

Participants were previously treated adult and adolescent (≥ 12 years) patients with severe hemophilia A (< 1 IU/dL [< 1%] endogenous FVIII or a documented genotype known to produce severe hemophilia A). Previous treatment (prophylaxis or on‐demand regimen) was defined as treatment with any recombinant and/or plasma‐derived FVIII product, or cryoprecipitate for ≥ 150 exposure days. Patients enrolled in Arm B were required to have had ≥ 12 bleeding episodes in the previous 12 months or ≥ 6 bleeding episodes in the previous 6 months prior to study enrollment. Patients with a history of inhibitors or with a positive inhibitor test result at screening were excluded.

### 2.3 Management of Bleeding Episodes

During the study, bleeding episodes were documented by the study participants in their electronic patient diary (bleeding episode data later reviewed by the investigator) and were treated with a single 50 IU/kg dose of efanesoctocog alfa. For minor/moderate bleeding episodes occurring within 2–3 days after a recent prophylactic dose, an initial 30 IU/kg dose could be used. If a bleeding episode did not improve, additional doses of 30 or 50 IU/kg every 2–3 days could be considered at the investigators' discretion.

### 2.4 Study Endpoints

The primary objective of the study was to evaluate the efficacy of efanesoctocog alfa as a prophylaxis treatment and the primary endpoint was annualized bleed rate (ABR) in Arm A. Secondary endpoints included ABR by type and location for prophylaxis treatment, number of injections and dose of efanesoctocog alfa used to treat a bleeding episode (defined using a standardized definition based on the International Society on Thrombosis and Haemostasis [ISTH] criteria), and assessment of response to efanesoctocog alfa treatment of individual bleeding episodes defined by ISTH criteria (ISTH 4‐point response scale per study arm and treatment regimen) [21]. Bleeding episodes were self‐reported by the participants and were not clinically confirmed.

### 2.5 Statistical Analysis

ABR in Arm A was estimated using a negative binomial model with the total number of treated bleeding episodes during the efficacy period as the response variable and log‐transformed efficacy period duration (in years) as an offset variable. ABRs, the number and location of treated bleeding episodes, and dose and number of efanesoctocog alfa administrations to resolve bleeding episodes are presented descriptively. Data are based on treated bleeding episodes in the full analysis dataset during the efficacy period, defined as the sum of all intervals of time during which patients are treated with efanesoctocog alfa according to the study arms and treatment regimen, excluding periods of pharmacokinetic evaluation, surgery/rehabilitation, and large injection intervals (> 28 days).

## 3 Results

### 3.1 Study Participants

In total, 159 patients were enrolled (Arm A, *n* = 133 and Arm B, *n* = 26) in XTEND‐1. All participants received ≥ 1 dose of efanesoctocog alfa. A total of 149 (94%) participants completed the study and 10 (6%) prematurely discontinued. The patients were mostly male (99%; 1 female was enrolled in Arm A), and the mean (SD) age was 35.4 (15.1) years (Table 1); 25 (16%) patients were between 12 and 17 years of age (all in Arm A). Baseline disease characteristics were representative of an adult and adolescent population with severe hemophilia A (Table 1). Five participants (3%) had a family history of inhibitors; no participant developed inhibitors during the XTEND‐1 study [19].

**TABLE 1:** Participant demographics and disease characteristics.

	Arm A prophylaxis (*n* = 133)	Arm B on‐demand and prophylaxis (*n* = 26)	Overall (*n* = 159)
Age, years			
Mean (SD)	33.9 (15.3)	42.8 (11.7)	35.4 (15.1)
Sex, *n* (%)			
Male	132 (99.2)	26 (100)	158 (99.4)
Female	1 (0.8)	0	1 (0.6)
Type of hemophilia treatment products administered throughout life, *n* (%)^a^			
*n*	127	26	153
FVIII plasma derived	83 (65.4)	24 (92.3)	107 (69.9)
FVIII recombinant	106 (83.5)	8 (30.8)	114 (74.5)
FVIII cryoprecipitate	32 (25.2)	9 (34.6)	41 (26.8)
Non‐FVIII products	25 (19.7)	2 (7.7)	27 (17.6)
Antifibrinolytic agents	15 (11.8)	2 (7.7)	17 (11.1)
Desmopressin/DDAVP	1 (0.8)	0	1 (0.7)
Emicizumab	6 (4.7)	0	6 (3.9)
Other	4 (3.1)	0	4 (2.6)
Prestudy regimen			
Prophylaxis, *n* (%)	133 (100)	1 (3.8)	134 (84.3)
On‐demand, *n* (%)	0	25 (96.2)	25 (15.7)
Time on pre‐study regimen, *n* (%)			
*n*	132	26	158
< 6 months	7 (5.3)	2 (7.7)	9 (5.7)
6–12 months	19 (14.4)	1 (3.8)	20 (12.7)
> 12 months	106 (80.3)	23 (88.5)	129 (81.6)
Number of bleeds in the 12 months prior to study enrollment, mean (SD)	3.2 (5.4)	35.7 (22.2)	8.3 (15.5)
Number of joint bleeds in the 12 months prior to study enrollment, mean (SD)	2.3 (4.5)	27. 4 (18.6)	6.0 (12.1)
Number of spontaneous joint bleeds in the 12 months prior to study enrollment, mean (SD)^b^	1.3 (2.5)	20.9 (14.2)	4.1 (9.0)
Target joints at baseline, ≥ 1, *n* (%)^c^	26 (19.5)	23 (88.5)	49 (30.8)
HJHS score at baseline, mean (SD)	18.1 (18.4)	26.3 (13.2)	—

*Note*: Some demographics have been previously reported. From *New England Journal of Medicine*. Von Drygalski, et al. Efanesoctocog Alfa prophylaxis for patients with severe hemophilia A. 388:310–318. Copyright © 2024 Massachusetts Medical Society. Reprinted with permission from Massachusetts Medical Society [19].

^a^ Participants may be counted in more than 1 category.

^b^ Retrospectively reported.

^c^ Reported by the investigator at enrollment.

### 3.2 Bleeding Episodes

During the 12‐month study duration of Arm A, the mean (standard deviation [SD]) efficacy period was 47.6 (8.77) weeks. Mean (SD) overall ABR in Arm A was 1.10 (1.92) for all bleeding episodes and 0.71 (1.43) for treated bleeding episodes. Mean (SD) spontaneous ABR, traumatic ABR, and joint ABR were 0.29 (0.73), 0.36 (0.83), 0.52 (1.09), respectively. Bleeding event rates were stable throughout the Arm A study duration (Figure S1).

During the 6‐month on‐demand treatment period in Arm B, the mean (SD) overall ABR was 22.22 (7.94) for all bleeding episodes and 21.42 (7.41) for treated bleeding episodes. During the prophylaxis period, the mean (SD) ABR in Arm B was 0.84 (1.61) for all bleeding episodes and 0.69 (1.35) for treated bleeding episodes. Mean (SD) spontaneous ABR, traumatic ABR, and joint ABR were 0.45 (1.13), 0.15 (0.78), and 0.61 (1.33), respectively.

A total of 56% of participants in Arm A experienced zero bleeding episodes overall and 65% had zero treated bleeding episodes during the study (Table 2). All participants in Arm B experienced bleeding episodes during on‐demand treatment; during the prophylaxis period in Arm B (mean [SD] efficacy period 22.9 [6.00] weeks), 73% of participants experienced zero bleeds and 77% experienced zero treated bleeds. During the study, a total of 422 bleeding episodes were reported by participants, of which 362 were treated. Most of these bleeding episodes (278/422 [66%] of all bleeding episodes and 268/362 [74%] of treated episodes) occurred in the 26 participants in Arm B during the on‐demand treatment period (Table 2). Of the 268 treated bleeding episodes during the on‐demand period of Arm B, 197 (74%) were spontaneous, 62 (23%) were traumatic, and 9 (3%) were of unknown etiology. The rates of treated spontaneous and traumatic bleeding episodes were low in Arm A. The majority (*n* = 107; 81%) of participants in Arm A had zero treated spontaneous bleeding episodes (Table 2). In Arm B during the prophylaxis treatment period, a total of 8 treated bleeding episodes occurred in 6/26 participants (Table 2); 5 treated bleeding episodes were spontaneous (4 of which occurred in joints), 2 were traumatic, and 1 was of unknown etiology. Only 1 spontaneous bleed occurred in a resolved target joint.

**TABLE 2:** Summary of ABRs and bleeding episodes in Arm A and Arm B.

	Arm A prophylaxis (*n* = 133)	Arm B on‐demand (*n* = 26)	Arm B prophylaxis (*n* = 26)
Mean (SD) duration of efficacy period, weeks	47.6 (8.8)	25.1 (1.1)	22.9 (6.0)
All bleeding episodes (treated and untreated)			
Total number of bleeding episodes	134	278	10
Participants with zero ABR, *n* (%)	75 (56.4)	0	19 (73.1)
Participants with ABR of > 0–5, *n* (%)	53 (39.8)	0	6 (23.1)
Participants with ABR of > 5–10, *n* (%)	3 (2.3)	1 (3.8)	1 (3.8)
Participants with ABR of > 10–20, *n* (%)	2 (1.5)	10 (38.5)	0
Participants with ABR > 20, *n* (%)	0	15 (57.7)	0
Treated bleeding episodes			
Participants with zero treated bleeding episodes, *n* (%)^a^, ^b^			
Total	86 (64.7)	0	20 (76.9)
Spontaneous	107 (80.5)	1 (3.8)	22 (84.6)
Traumatic	103 (77.4)	8 (30.8)	25 (96.2)
Joint	96 (72.2)	0	21 (80.8)
Spontaneous joint	112 (84.2)	1 (3.8)	23 (88.5)
Total number of treated bleeding episodes, *n*	86	268	8
Type of treated bleeding episodes, *n* (%)			
Spontaneous	33 (38.4)	197 (73.5)	5 (62.5)
Traumatic	45 (52.3)	62 (23.1)	2 (25)
Unknown	8 (9.3)	9 (3.4)	1 (12.5)
Location of treated bleeding episodes, *n* (%)^c^			
Joint	61 (56.5)	219 (81.1)	7 (87.5)
Muscle	25 (23.1)	27 (10.0)	0
Internal	7 (6.5)	5 (1.9)	0
Skin/mucosa	15 (13.9)	18 (6.7)	1 (12.5)
Unknown location	0	1 (0.3)	0

Abbreviations: ABR, annualized bleed rate; IQR, interquartile range; PK, pharmacokinetic; SD, standard deviation.

^a^ Percentages are based on the number of participants in each study arm and treatment regimen with an evaluable efficacy period. Efficacy period was defined as the sum of all intervals of time during which participants are treated with efanesoctocog alfa according to the study arms and treatment regimen, excluding periods of PK evaluation, surgery/rehabilitation, and large injection intervals.

^b^ This does not include type unknown.

^c^ A single bleeding episode could occur at more than 1 location and so the total number of bleeding episodes by location differs from the overall total number of bleeding episodes. Percentages are based on the total number of bleeding episodes by location.

**FIGURE 1:**
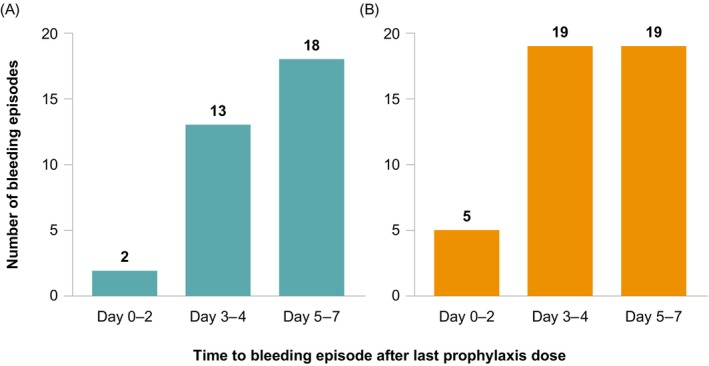
Time after the last prophylaxis dose to treated (A) spontaneous bleeding episode or (B) traumatic^a,b^ bleeding episode in Arm A. Bleeding episodes were reported by the study participants and were not adjudicated. ^a^One traumatic bleeding episode (not shown) occurred 13 days after the last prophylaxis dose. ^b^One traumatic bleeding episode (not shown) occurred during screening and thus had no prior prophylaxis dose. [Color figure can be viewed at wileyonlinelibrary.com]

Overall in Arm A, 86 treated bleeding episodes occurred in 47/133 patients during the 12‐month treatment period, of which 45 (52%) were traumatic, 33 (38%) were spontaneous (including 25 spontaneous joint bleeds), and 8 (9%) were of unknown etiology. In both Arm A and Arm B, the most common locations for treated bleeding episodes were joints and muscles. In Arm A, 18 treated spontaneous bleeding episodes occurred 5–7 days after the last prophylaxis dose, 13 occurred 3–4 days after the last dose, and 2 occurred 0–2 days after the last dose (Figure 1). For the 2 spontaneous bleeds within 0–2 days following dosing, neither participant had target joints at baseline and Hemophilia Joint Health Scores (HJHS) scores at baseline were 13 and 32 (HJHS range is 0–124, with 0 being optimum), respectively. Based on available narratives, 1 of these 2 participants experienced an ankle bleed after a long period of walking. Overall, 26 patients in Arm A had a total of 33 treated spontaneous bleeding episodes, with 21 of those participants (81%) experiencing a single spontaneous bleeding episode. For traumatic bleeding episodes, 19 each occurred in the 3–4 days and 5–7 days following the last prophylaxis dose, and 5 occurred 0–2 days after the last prophylaxis dose (Figure 1).

### 3.3 Treatment of Bleeding Episodes

A single injection was sufficient to resolve 97% (350/362) of treated bleeding episodes overall (Table 3); a total of 11 (3%) bleeding episodes in 7 participants were treated with 2 injections and 1 (0.3%) bleeding episode (a traumatic bleed at joint, muscle, and skin/mucosa due to an arm fracture) was treated with 3 injections. No bleeding episodes were treated with more than 3 injections. The median (IQR) efanesoctocog alfa dose per injection was 50.9 (50.0–51.5) IU/kg and the median (IQR) total dose was 50.9 (50.0–51.9) IU/kg per bleeding episode.

**TABLE 3:** Summary of bleed treatment in Arm A and Arm B.

	Arm A prophylaxis (*n* = 133)	Arm B on‐demand (*n* = 26)	Arm B prophylaxis (*n* = 26)	Overall (*N* = 159)
Injections required to resolve a bleed, *n* (%)^a^				
1	81 (94.2)	261 (97.4)	8 (100)	350 (96.7)
2	4 (4.7)	7 (2.6)	0	11 (3.0)
3	1 (1.2)^b^	0	0	1 (0.3)^b^
Dose per injection (IU/kg)				
Mean (SD)	46.72 (9.22)	50.58 (2.47)	45.74 (9.52)	49.56 (5.42)
Median (IQR)	50.85 (47.17–52.20)	50.90 (50.00–51.43)	50.30 (40.00–51.42)	50.85 (50.00–51.51)
Total dose per bleeding episode (IU/kg)				
Mean (SD)	49.95 (16.63)	51.74 (6.56)	45.74 (9.52)	51.18 (10.00)
Median (IQR)	51.00 (50.00–52.42)	50.93 (50.03–51.47)	50.30 (40.00–51.42)	50.93 (50.00–51.85)
Number of evaluable bleeds	73	255	6	334
Response to bleed treatment, *n* (%)^c^, ^d^				
Excellent	39 (53.4)	195 (76.5)	5 (83.3)	239 (71.6)
Good	21 (28.8)	56 (22.0)	1 (16.7)	78 (23.4)
Moderate	10 (13.7)	4 (1.6)	0	14 (4.2)
None	3 (4.1)	0	0	3 (0.9)^e^

Abbreviations: ISTH, International Society on Thrombosis and Haemostasis; IQR, interquartile range; PK, pharmacokinetic; SD, standard deviation.

^a^ Percentages are based on the total number of bleeding episodes in the efficacy period. Efficacy period was defined as the sum of all intervals of time during which participants are treated with efanesoctocog alfa according to the study arms and treatment regimen, excluding periods of PK evaluation, surgery/rehabilitation, and large injection intervals.

^b^ Bleed with 3 injections was a traumatic bleed at joint, muscle, and skin/mucosa due to arm fracture.

^c^ Percentages based on the total number of evaluable bleeds per column.

^d^ Patient assessment of the response to bleed treatment evaluated with the 4‐point ISTH scale. “None” meaning no or minimal improvement, or condition worsened, within approximately 8 h after the initial injection.

^e^ Of the 3 responses to bleed treatment recorded as “None,” one response was reported following a fall with multiple traumas and one response was reported following a fight and incorrectly linked to a bleeding episode that occurred before study baseline. Therefore, these responses may not be appropriate.

A total of 334 injections had a participant evaluation of response to treatment. Overall, 95% (317/334) of the injections had a response rated as excellent or good (Table 3). Physician global assessment of participant response to treatment was rated as excellent for 96% and 93% of all visits during prophylaxis in Arms A and B, respectively.

### 3.4 Consumption

The mean (SD) and median (IQR) annualized consumption per participant during the efficacy period was 3131.8 (4113.2) IU/kg and 2757.0 (2705.7–2805.8) IU/kg for participants in Arm A, respectively. Mean (SD) and median (IQR) consumption for participants in Arm B was 1135.3 (404.9) IU/kg and 1212.3 (769.9–1382.2) during on‐demand treatment and 2751.5 (88.3) IU/kg and 2737.5 (2718.7–2818.4) during prophylaxis treatment, respectively.

The mean (SD) and median (IQR) average weekly prophylactic dose of efanesoctocog alfa was 51.3 (2.2) and 51.3 (50.4–52.1) for participants in Arm A, and 51.0 (1.3) and 50.9 (50.3–51.6) for participants in Arm B during prophylaxis treatment, respectively.

## 4 Discussion

In the XTEND‐1 study, efanesoctocog alfa provided highly effective prevention and treatment of bleeding episodes in previously treated patients with severe hemophilia A. The bleed event rate in participants treated with once‐weekly efanesoctocog alfa prophylaxis was low and consistent throughout the duration of the study, indicating once‐weekly efanesoctocog alfa prophylaxis was protective against bleeds. A single 50 IU/kg dose of efanesoctocog alfa was sufficient to resolve 97% of bleeding episodes regardless of bleed type and location in participants receiving prophylaxis or on‐demand treatment.

Suboptimal prevention of bleeds in the long‐term can lead to progression of joint disease with permanent impacts on physical health and quality of life [7, 22]. There is increasing evidence that a FVIII trough level of 1% is not sufficient to prevent all bleeds, clinical, or subclinical [1, 9, 14]. Achieving higher FVIII levels over longer periods of time could potentially lead to an almost bleed‐free status in patients with hemophilia [9, 22]. Efanesoctocog alfa, a high‐sustained FVIII therapy, overcomes the VWF‐imposed limit on FVIII half‐life to provide a half‐life 3‐ to 4‐fold longer than SHL and EHL FVIII products (octocog alfa and rurioctocog alfa pegol) [18]. Published findings from the XTEND‐1 trial in adults and adolescents with severe hemophilia A showed that efanesoctocog alfa had a geometric mean half‐life of 47.0 h (95% confidence interval, 42.3–52.2) and maintained FVIII levels in the normal to near‐normal range of > 40% for 4 days and 15% by the end of the week [19].

The results of the present analysis showed that most of the bleeding episodes reported by participants in XTEND‐1 occurred in participants in Arm B during the on‐demand treatment period as compared with prophylaxis periods, which is expected. The median (IQR) ABR for all bleeding episodes in Arm A was 0.00 (0.00–1.15) and in Arm B during prophylaxis was 0.00 (0.00–0.00), which is lower than reported for EHL FVIII products [23, 24, 25]. The proportion of bleeds treated with 1 infusion was 97%, which is higher than reported for EHL FVIII products. A long‐term continuation study of rurioctocog alfa pegol found that the overall median (IQR) ABR in all participants receiving prophylaxis was 1.62 (0.52–2.83), and 89% of bleeds were treated with 1 or 2 infusions [23]. The PROTECT VIII extension study found that the median (IQR) total ABR in all patients receiving damoctocog alfa pegol prophylaxis was 1.49 (0.36–4.80) [24], and 81% of bleeds were treated with 1 infusion [25]. In XTEND‐1, approximately two‐thirds of participants receiving once‐weekly efanesoctocog alfa experienced zero treated bleeds during prophylactic treatment across both arms. The bleed event rate was consistent throughout the duration of the study in Arm A, showing that weekly prophylaxis with efanesoctocog alfa provided immediate, effective protection against bleeds. Furthermore, bleed rates were overall low across both type (spontaneous or traumatic) and location (joint, muscle, internal, or skin/mucosa) of bleeding in participants receiving prophylaxis. In Arm A, most (18/33) of the reported spontaneous bleeding episodes occurred 5–7 days after the last prophylaxis dose; 13 occurred 3–4 days after the last dose, and 2 occurred 0–2 days after the last dose. A limitation of this study is that occurrence and details of bleeding episodes (type, location, and response to treatment) were self‐reported by participants and were not clinically confirmed, which is a general limitation for most trials in hemophilia.

Severe hemophilia has a substantial impact on everyday life, and even EHL products may present a significant treatment burden. New treatments such as non‐factor therapies have been developed to address this issue; however, breakthrough bleeds can occur that require treatment with factor replacement. For example, severe muscle bleeds have been reported in children and young adults without inhibitors on emicizumab prophylaxis. The treatment of these bleeds required extensive and prolonged treatment with factor replacement and hospitalization was required in some cases. A median of 12 doses and 11 factor exposure days for resolution was reported by Batsuli et al. and all patients were hospitalized and required up to 10 factor exposure days as reported by Garcia et al. [26, 27] As well as providing effective bleed protection with a weekly dosing interval, efanesoctocog alfa provided efficient treatment of bleeding episodes. In the participants who experienced bleeding episodes, 97% of treated bleeding episodes were resolved with a single 50 IU/kg dose of efanesoctocog alfa in both treatment arms. The vast majority of participants who experienced a bleeding episode assessed the response to treatment as excellent or good. The high‐sustained FVIII activity provided by efanesoctocog alfa can reduce the treatment burden and improve physical health and quality of life outcomes for people with hemophilia [19], potentially being an attractive option for those with hemophilia who have historically been reticent to dose prophylactically (i.e., individuals who have preferred to remain on an on‐demand regimen). All participants who had exit interviews preferred efanesoctocog alfa prophylaxis to their prior treatment [19].

The findings here show that once‐weekly efanesoctocog alfa provided highly effective bleed protection through high‐sustained FVIII levels in patients with severe hemophilia A.

## Ethics Statement

XTEND‐1 was conducted in accordance with international guidelines, including the Declaration of Helsinki, and International Council for Harmonization guidelines for Good Clinical practice, as well as all applicable laws, rules, and regulations.

## Consent

Patients/guardians provided written informed consent, which was obtained prior to enrollment in the study.

## Conflicts of Interest

ACW has received fees from Hemab, Sobi, Takeda, Sanofi, Genentech, Spark, Novo Nordisk, BioMarin, and Bayer for consultation and involvement in advisory boards. She has received research funding from Pfizer, Novo Nordisk, Takeda, and Sanofi. SM has received speaker fees from CSL Behring, Novo Nordisk, Roche, Sobi, and Takeda and fees for consultation and involvement in advisory boards from Baxalta, CSL Behring, Novo Nordisk, Octapharma, Roche, Takeda, and Sobi. LB and LAF are employees of Sobi; may hold shares and/or stock options in the company. GN and AW are employees of Sanofi; may hold shares and/or stock options in the company. AT has received speaker fees from Bayer, CSL Behring, GSK, Novo Nordisk, and Werfen.

## Supporting information


Data S1.

Once weekly efanesoctocog alfa (50 IU/kg) prophylaxis provided highly effective bleed protection to previously treated adults and adolescents ≥12 years of age with severe hemophilia A. Efanesoctocog alfa also provided effective treatment of bleeding episodes, with most bleeding episodes being resolved with a single injection (50 IU/kg).

## Data Availability

Qualified researchers may request access to patient‐level data and related documents (including, eg, the clinical study report, study protocol with any amendments, blank case report form, statistical analysis plan, and dataset specifications). Patient‐level data will be anonymized, and study documents will be redacted, including to protect the privacy of trial participants. Further details on Sanofi's data sharing criteria, eligible studies, and process for requesting access can be found at: https://www.vivli.org/.
