# Supplementary material for: Treatment of Bleeding Episodes With Efanesoctocog Alfa in Previously Treated Patients With Severe Hemophilia A in the Phase 3 XTEND‐1 Study
Source: Am J Hematol. 2025 Feb 10;100(5):813–20. doi: 10.1002/ajh.27603 (PMC11966352; doi:10.1002/ajh.27603)
Supplement: Supplementary file 1 — Data S1. [file AJH-100-813-s001.docx]

# Supplementary Information

**Figure S1:** Bleeding event rate for treated bleeding episodes was stable throughout the study duration in Arm A^a^


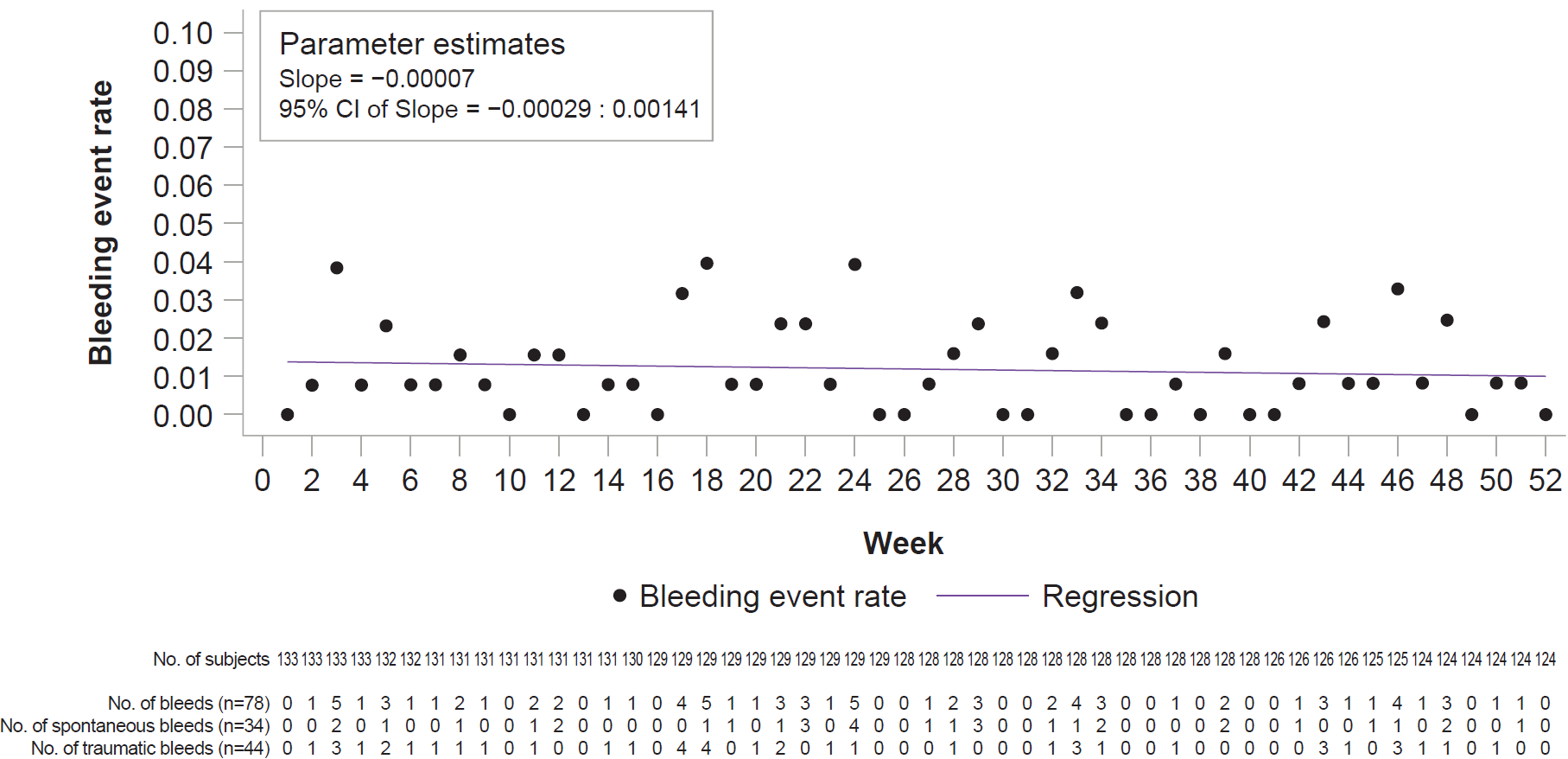


CI, confidence interval.

^a^Analysis includes treated bleeds with non-missing bleed dates (n=78), spontaneous bleeds (n=34), and traumatic bleeds (n=44) up to Week 52 in Arm A. Bleeding episodes are mapped to each week based on event date and time. Bleeding event rates are calculated as the number of bleeds per patient week. A linear regression model was used to determine the slope and 95% CI of the slope.
