# Supplementary material for: Treatment of Bleeding Episodes With Efanesoctocog Alfa in Previously Treated Patients With Severe Hemophilia A in the Phase 3 XTEND‐1 Study
Source: Am J Hematol. 2025 Feb 10;100(5):813–20. doi: 10.1002/ajh.27603 (PMC11966352; doi:10.1002/ajh.27603)
Supplement: Supplementary file 2 — Once weekly efanesoctocog alfa (50 IU/kg) prophylaxis provided highly effective bleed protection to previously treated adults and adolescents ≥12 years of age with severe hemophilia A. Efanesoctocog alfa also provided effective treatment of bleeding episodes, with most bleeding episodes being resolved with a single injection (50 IU/kg). [file AJH-100-813-s002.pdf]

XTEND-1: Phase 3, Multicenter Study (NCT04161495)

159 previously treated adults and adolescents (≥12 years old) with severe hemophilia A

ARM A (n=133)

Weekly prophylaxis efanesoctocog alfa 50 IU/kg

ARM B (n=26)

On-demand efanesoctocog alfa 50 IU/kg → Weekly prophylaxis efanesoctocog alfa 50 IU/kg

Baseline → **Week 26** → **Week 52**

Bleeding Episode Treatment During XTEND-1

- A single 50 IU/kg dose of efanesoctocog alfa was given for a bleeding episode
  - Bleeding episodes that did not improve could be treated with an additional dose of 30 or 50 IU/kg every 2–3 days
- Minor/moderate bleeding episodes within 2–3 days of a recent prophylaxis dose could be treated with an initial 30 IU/kg dose

This analysis further evaluates bleeding episodes observed during XTEND-1, as well as efficacy of efanesoctocog alfa in treatment of bleeding episodes

74% n=268 of the 362 treated bleeding episodes that occurred in XTEND-1 were during the on-demand period of Arm B

65% n=86 of participants in Arm A had **0** treated bleeding episodes

81% n=107 of participants in Arm A had **0** treated spontaneous bleeding episodes

Most (n=18) **spontaneous bleeds** occurred **5–7 days** after the last prophylaxis dose

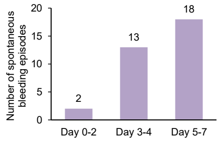

19 **traumatic bleeds** each occurred **3–4** and **5–7 days** after the last dose

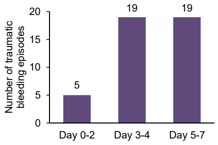

Time to bleeding episode after last prophylaxis dose

A **single injection** of efanesoctocog alfa (50 IU/kg) was **sufficient to resolve 97%** (n=350) of treated bleeding episodes

95% of the injections with an evaluation had a response **rated as excellent or good**

Efanesoctocog alfa provided highly effective bleed protection through high-sustained factor VIII activity as well as highly effective treatment of bleeding episodes in patients with severe hemophilia A
